# Clinical Protocol for a Longitudinal Cohort Study Employing Systems Biology to Identify Markers of Vaccine Immunogenicity in Newborn Infants in The Gambia and Papua New Guinea

**DOI:** 10.3389/fped.2020.00197

**Published:** 2020-04-30

**Authors:** Olubukola T. Idoko, Kinga K. Smolen, Oghenebrume Wariri, Abdulazeez Imam, Casey P. Shannon, Tida Dibassey, Joann Diray-Arce, Alansana Darboe, Julia Strandmark, Rym Ben-Othman, Oludare A. Odumade, Kerry McEnaney, Nelly Amenyogbe, William S. Pomat, Simon van Haren, Guzmán Sanchez-Schmitz, Ryan R. Brinkman, Hanno Steen, Robert E. W. Hancock, Scott J. Tebbutt, Peter C. Richmond, Anita H. J. van den Biggelaar, Tobias R. Kollmann, Ofer Levy, Al Ozonoff, Beate Kampmann

**Affiliations:** ^1^Vaccines and Immunity Theme, Medical Research Council Unit the Gambia at London School of Hygiene and Tropical Medicine, Fajara, Gambia; ^2^Precision Vaccines Program, Division of Infectious Diseases, Boston Children's Hospital, Boston, MA, United States; ^3^CIH LMU Center for International Health, Medical Center of the University of Munich (LMU), Munich, Germany; ^4^The Vaccine Centre, London School of Hygiene and Tropical Medicine, London, United Kingdom; ^5^Harvard Medical School, Boston, MA, United States; ^6^PROOF Centre of Excellence, Vancouver, BC, Canada; ^7^Department of Pediatrics, BC Children's Hospital, University of British Columbia, Vancouver, BC, Canada; ^8^Division of Medicine Critical Care, Harvard Medical School, Boston Children's Hospital, Boston, MA, United States; ^9^Department of Cardiology, Boston Children's Hospital, Boston, MA, United States; ^10^Wesfarmers Centre of Vaccines and Infectious Diseases, Telethon Kids Institute, University of Western Australia, Nedlands, WA, Australia; ^11^Papua New Guinea Institute of Medical Research, Goroka, Papua New Guinea; ^12^BC Cancer Agency, Vancouver, BC, Canada; ^13^Department of Medical Genetics, University of British Columbia, Vancouver, BC, Canada; ^14^Department of Pathology, Boston Children's Hospital, Boston, MA, United States; ^15^Department of Microbiology & Immunology, University of British Columbia, Vancouver, BC, Canada; ^16^Centre for Heart Lung Innovation, University of British Columbia, Vancouver, BC, Canada; ^17^Division of Respiratory Medicine, Department of Medicine, UBC, Vancouver, BC, Canada; ^18^Division of Pediatrics, School of Medicine, Perth Children's Hospital, University of Western Australia, Nedlands, WA, Australia; ^19^Broad Institute of MIT & Harvard, Cambridge, MA, United States

**Keywords:** markers, newborn, vaccine, immunogenicity, systems biology, OMICS

## Abstract

**Background:** Infection contributes to significant morbidity and mortality particularly in the very young and in low- and middle-income countries. While vaccines are a highly cost-effective tool against infectious disease little is known regarding the cellular and molecular pathways by which vaccines induce protection at an early age. Immunity is distinct in early life and greater precision is required in our understanding of mechanisms of early life protection to inform development of new pediatric vaccines.

**Methods and Analysis:** We will apply transcriptomic, proteomic, metabolomic, multiplex cytokine/chemokine, adenosine deaminase, and flow cytometry immune cell phenotyping to delineate early cellular and molecular signatures that correspond to vaccine immunogenicity. This approach will be applied to a neonatal cohort in The Gambia (*N* ~ 720) receiving at birth: (1) Hepatitis B (HepB) vaccine alone, (2) Bacille Calmette Guerin (BCG) vaccine alone, or (3) HepB and BCG vaccines, (4) HepB and BCG vaccines delayed till day 10 at the latest. Each study participant will have a baseline peripheral blood sample drawn at DOL0 and a second blood sample at DOL1,−3, or−7 as well as late timepoints to assess HepB vaccine immunogenicity. Blood will be fractionated via a “small sample big data” standard operating procedure that enables multiple downstream systems biology assays. We will apply both univariate and multivariate frameworks and multi-OMIC data integration to identify features associated with anti-Hepatitis B (anti-HB) titer, an established correlate of protection. Cord blood sample collection from a subset of participants will enable human *in vitro* modeling to test mechanistic hypotheses identified *in silico* regarding vaccine action. Maternal anti-HB titer and the infant microbiome will also be correlated with our findings which will be validated in a smaller cohort in Papua New Guinea (*N* ~ 80).

**Ethics and Dissemination:** The study has been approved by The Gambia Government/MRCG Joint Ethics Committee and The Boston Children's Hospital Institutional Review Board. Ethics review is ongoing with the Papua New Guinea Medical Research Advisory Committee. All de-identified data will be uploaded to public repositories following submission of study output for publication. Feedback meetings will be organized to disseminate output to the study communities.

**Clinical Trial Registration**: Clinicaltrials.gov Registration Number: NCT03246230

## Background and Rationale for the Study

Infection remains a major cause of morbidity and mortality accounting for over 30% of global deaths occurring each year in children under the age of 5 years (1). The burden of infectious disease is highest among the very young and in low- and middle-income countries (1, 2). In 2017, 5.4 million deaths occurred in children under the age of five accounting for 38.9 deaths per thousand live births (2). Roughly 2.5 million (47%) of these under five deaths occurred in the first 28 days of life (1). The leading causes of under-five mortality in 2016 were complications of prematurity (18%), pneumonia (16%) intrapartum related events (12%), congenital anomalies (9%), diarrhea (8%), neonatal sepsis (7%), and malaria (5%) (1). Thus, infectious disease remains a leading cause of mortality and indeed morbidity during the most vulnerable period of life.

Immunization is a powerful and highly cost-effective approach to prevent infection (3–5). It is estimated that for every public dollar spent on immunization, there is a $44 return on investment (6), and that vaccines have contributed to saving over 20 million lives and $350 billion between 2001 and 2017 in 73 low and middle income countries alone (6). Vaccines are thought to save 2–3 million lives each year worldwide (7) and have contributed to disease eradication (8–10) with prospects for eradicating another disease in sight (11–13). However, there are few vaccines specifically licensed for use at the extremes of age partly because little is known regarding the molecular pathways by which vaccines induce protection, particularly in the “developing” immune system of the very young (14).

**Figure 1:**
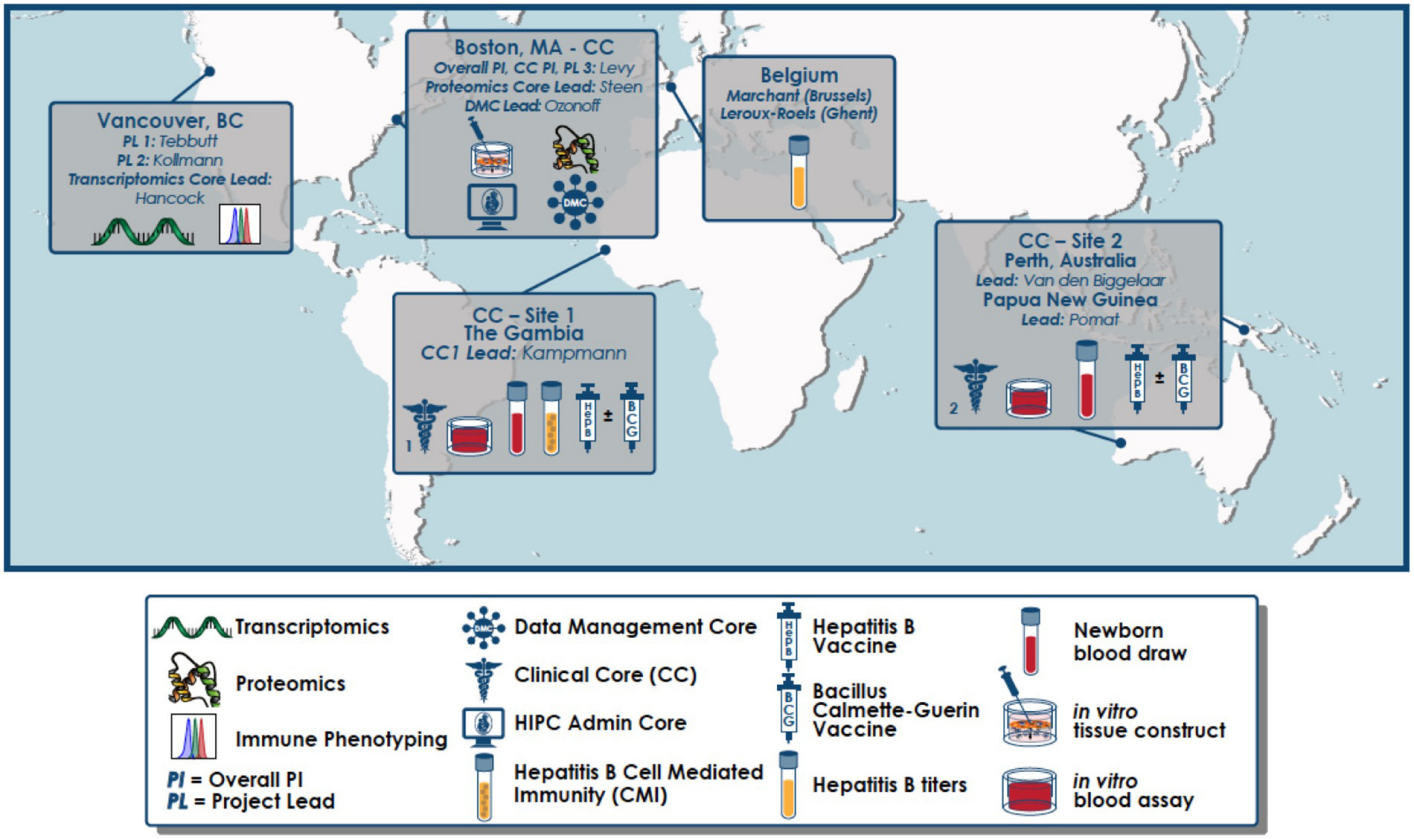
Geographical distribution of partnering sites for the EPIC-002 study. An overarching administrative core, clinical core (CC), data management core (DMC), as well as an *in vitro* vaccine modeling project are based at Boston Children's Hospital (Boston, MA). Clinical Core Sites are located in The Gambia (West Africa) and Papua New Guinea (Australasia). End-point assays are conducted in The Gambia (whole blood assay and cell mediated immunity), PNG (whole blood assay), University of British Columbia (flow cytometry and RNASeq), BCH (multiplex cytokines/chemokines, plasma proteomics, *in vitro* modeling including WBA and tissue constructs) as well as the Center for Vaccinology (CEVAC; Ghent, Belgium; anti-hepatitis B surface antigen titres).

Systems biology, is a powerful approach to gain deep insight into biology and has increasingly been applied to vaccinology to obtain insights into vaccine protection (15). However, these powerful techniques have not been applied to the most vulnerable: newborns in resource poor settings (16). To meet this need, an international group of academic biomedical centers have partnered to form the Expanded Program on Immunization Consortium (EPIC) (Figure 1) partnering to utilize systems biology to unravel the complex relationships between vaccine immunogenicity via early, vaccine-induced transcriptomic, metabolomic, proteomic, multiplex cytokine/chemokine, adenosine deaminase and immune cell phenotype (“OMIC”) signatures. With funding from the United States National Institute of Health (NIH), through National Institute of Allergy and Infectious Diseases (NIAID) U19 Initiative for the Human Immune Project Consortium (HIPC), the study described below will be conducted to explore these complex relationships.

Previous EPIC pilot studies (EPIC-001) conducted at the Medical Research Council Unit The Gambia at London School of Hygiene and Tropical Medicine (MRCUG at LSHTM) and the Papua New Guinea Institute of Medical Research (PNGIMR) between 2015 and 2017 demonstrated the feasibility of measuring robust and cogent “OMIC” readouts from small volume blood samples to obtain “OMIC” signatures (17). Based on the pilot data, the current EPIC research project will focus on the immunogenicity of Hepatitis B (HepB) vaccine for which a clear correlate of protection exists (i.e., anti-hepatitis B surface antigen antibody (Ab) titres) (18, 19). Cell mediated immunity (CMI) though not validated as a correlate of protection but which appears to play a role in long-term immunity (post-primary series titres of 10 mIU/ml or greater have been shown to correlate with the induction of memory T helper-and B-cell responses) (20), will also be assessed. In addition, we will examine if concomitant vaccination with Bacille Calmette Guérin (BCG) can modulate the response to HepB vaccine. Both vaccines are recommended for routine use at birth in these countries and in similar settings.

The HepB vaccine is safe, immunogenic and highly effective and is on the list of Expanded Programme on Immunization (EPI) recommended childhood vaccines. The first dose is recommended on the day of birth to prevent vertical and horizontal transmission of Hepatitis B virus (HBV) (18). The HepB vaccine has one of the best-characterized serologic correlates of protection (CoP), and is the only clear CoP for any neonatal vaccine (18). First described in The Gambia, this CoP is the lower limit of the peak Ab response measured >1 month after the primary series (3 or 4 doses), defined as an anti-Hepatitis B surface antigen (anti-HBs) Ab level of >10 mIU/mL (18, 19). Importantly, while an anti-HBs threshold of >10 mIU/ml represents the minimal level for protection from infection, a relationship exists in direct quantitative correlation between Ab level and duration of protection (i.e., the higher the anti-HBs titer the better and longer protection) (19). In addition, there is evidence to suggest that the higher the titer after the first dose of HepB vaccine, the higher the titer after the last dose, and persistence (18, 19). This implies that variability in response to the first dose (21) of HepB vaccine may predict long-term immunogenicity (18). Substantial quantitative inter-participant variation in absolute anti-HBs levels are the norm after each dose of HepB vaccine as with many other vaccines. Such inter-participant variability represents a key ingredient for meaningful analyses using a systems vaccinology approach (21).

The neonatal HepB vaccine immune response has been reported to be altered by co-administration of BCG, which is co-administered as part of the standard EPI, and likely to perturb the immune response to HepB vaccine *in vivo* (22, 23). Maternal antibodies to HepB have also been documented to impair the post vaccination immune response to HepB vaccine in newborns (18, 24, 25). The mechanisms that underlie HepB vaccine-induced immunogenicity in newborns, including Ab and CMI, are incompletely characterized particularly as it relates to variations in response.

Given the many advantages, HepB vaccine represents an ideal model in which to decipher what governs immunogenicity in early life. Similar reasoning regarding HepB vaccine as a model for systems biology have led to deciphering of mechanisms relevant for the immune response in older adults (26). This project will study the systems biology responses of newborns receiving HepB vaccination with and without concomitant BCG vaccine compared to a group of infants receiving both vaccines later in the first week of life to allow us to observe any changes occurring over the first week of life as a result of immune ontogeny rather than induced by vaccination. *In vitro* tissue constructs will also be set up to model the *in vivo* responses and maternal antibodies will be assayed to enable correlation with the infant responses.

The ability to show a correlation between age-specific molecular patterns (“signatures”) and vaccine-mediated protection (our main outcome measure) should accelerate the development and optimization of vaccines against childhood infections of major global health importance.

## Study Questions, Objectives

The overall goal of the project is the characterization of vaccine-specific “OMIC” signatures that correlate with vaccine-type specific immunogenicity in human newborns.

The EPIC-002 study will characterize vaccine-induced “OMIC” signatures and their relationship to HepB vaccine CoP and characterization of the impact of BCG and maternal antibodies (MatAbs), thereby informing development of vaccines optimized for early life immunization.

Specific Objectives:

1. Measure adaptive immune responses to HepB vaccine, enabling correlation of *in vivo* “OMIC” signatures and *in vitro* vaccine Modeling assays with established correlates of protection.
2. Characterize the pre-vaccine “OMIC” and immune *in vivo* signatures that may predict immunogenicity of HepB vaccine in human newborns.
3. Characterize the impact of HepB vaccine with or without BCG on neonatal “OMIC” and immune *in vivo* signatures that predict immunogenicity of HepB vaccine.
4. Measure maternal antibodies (MatAbs) to HBV in relation to vaccine-induced neonatal and infant “OMIC” vaccine signatures and adaptive responses.
5. Interrogate functional correlations identified *in silico* using novel human *in vitro* platforms.
6. Validate identified “OMIC” signatures in a distinct and independent newborn cohort recruited from Papua New Guinea (PNG).

## Study Design

The program consists of two neonatal cohort studies. A longitudinal neonatal cohort study will be conducted in The Gambia with a total follow-up time of roughly 5 months that serves as the program's core study. Following completion and analysis of The Gambian study, a validation cohort study will be conducted in Papua New Guinea to validate the key findings of the Gambian study. The two studies will harmonize procedures and protocols.

Participants will be recruited within the first 24 h of life into one of four broad groups as detailed below to receive either HepB vaccine alone, BCG vaccine alone or both HepB and BCG vaccines at this time point. The fourth group will have the birth doses of vaccines deferred till a time point within the first week of life. All infants will receive the recommended birth dose of polio vaccine a maximum of 10 days post-vaccination depending on the group assignment.

Two mL of venous blood will be drawn from each participant at the point of recruitment to assess “OMIC” responses. Each participant will have a maximum of two blood draws within the first week of life. In the core cohort in The Gambia, infants will be randomized into three subgroups that will have the second blood sample collected at 1, 3, or 7 days following the initial sampling. The cohort in The Gambia therefore consists of 12 different groups (Table 1). For the PNG cohort, all infants will have the second blood draw on the same day of life: analysis of The Gambia cohort will inform whether this will be at day 1, 3, or 7 of life. Subsequent visits and blood draws are as detailed in the Clinical Cohort Table below (Table 1)

**Table 1:**
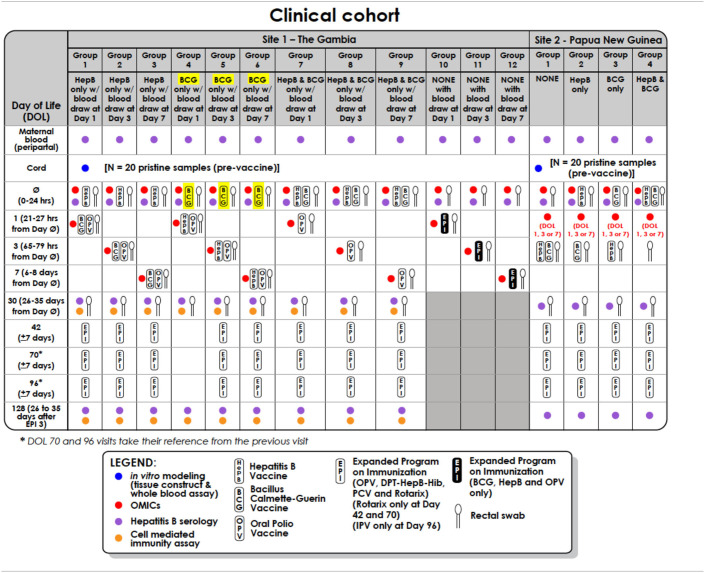
Clinical cohort table for infants recruited in The Gambia (discovery cohort) and Papua New Guinea (validation cohort).

*Table courtesy Kristin Johnson, Boston Children's Hospital*.

*In vitro* effects of the HepB vaccine and BCG vaccines will be studied in two *in vitro* assays using blood derived from the same study participants immunized *in vivo*: (a) whole blood assay (WBA) run at the recruitment sites on the day of sample collection and (b) in three-dimensional microphysiologic tissue constructs employing cryopreserved cord blood-derived mononuclear cells and plasma (27), enabling *in vitro* mechanistic interrogation of cellular and molecular signatures that correlate with anti-HepBsAg (anti-Hepatitis B surface antigen) titres *in vivo*.

Blood samples and rectal swabs for epigenetic and microbiome analysis will be collected and stored from participants in The Gambia. Maternal blood samples to enable correlation of maternal anti-HB Abs to infant responses will also be collected.

## Study Analysis Plan

This study aim is to identify “OMIC” signatures that correlate with HepB vaccine responses, as assessed by anti-HB titer (a well-established CoP), by leveraging both pre- and post-vaccine transcriptomic, proteomic, metabolomic, and cellular profiles. Our intention is to apply both univariate and multivariate frameworks to identify either a ranked list of molecular features associated with anti-HB titer or subsets of the molecular features best able to predict anti-HB titer, respectively. We plan to derive signatures in three distinct groups of newborns (corresponding to the distinct vaccination schedules) and compare across groups to gain a better understanding of the molecular basis of adjuvantation of HepB vaccine with BCG (given before, after or simultaneously). We further plan to derive robust multi-“OMIC” signatures correlating with effective HepB immunization by integrating across all available “OMIC” datasets. These signatures will then be validated in a distinct and independent newborn cohort recruited from PNG. Finally, the correlations identified by these various *in silico* analyses will be tested using the novel human *in vitro* platforms.

We will apply these methods to define baseline cellular and molecular pathways/hubs/nodes that predict vaccine immunogenicity and assess specific signatures *in vitro* that provide insight into vaccine mechanisms of action (MOA).

## Sample Size/Biostatistical Considerations

The cohort design included careful consideration of statistical power and sample size. Power calculations in the setting of traditional OMIC designs are well-documented (28) and we performed power calculations for OMIC platforms or flow cytometry based on preliminary data (unpublished data). Although we intended our tests of hypothesis to borrow strength across data types, little has been published on statistical power for integrative analyses or cross-platform comparisons, so this characteristic of our design was not taken into account when determining sample size. Power calculations were carried out using group sizes ranging between *n* = 30 or *n* = 60 per group, two-sided significance criterion (alpha) of 0.05, and a target 80% power to detect.

Since preliminary data for flow cytometry were not available, we performed a worst-case power analysis using a real-world published data on the most variable cell subpopulation where a significant difference was still identified comparing healthy, immunosuppressed, and transplant-tolerant individuals (29). This provided a lower bound of the minimum sample size required to detect a significant difference in cell sub-populations that could act as potential biomarkers. We found *n* = 30 would result in 80% power to detect a 2.3-fold difference in the relative abundance of a cell population, while *n* = 60 would result in 80% power to detect 1.8-fold difference, assuming a balanced design.

### OMIC Analyses

We considered power to detect correlations between biomarker signals of any type and the CoP. We assumed approximate normality, after data transformation (e.g., log-transformation) and calculated detectable Pearson coefficients for samples using group size *n* = 30 or *n* = 60. We concluded that our design offered 80% power to detect correlations between continuous measures as small as *r* = 0.25–0.48. Additional parameters were included when carrying out power calculations for the transcriptomic analysis to account for variable coverage [e.g., read depth across biosamples using RNASeqPower v1.10 (28)]. Here we considered power to detect differentially abundant transcripts between two groups and estimated within-group standard deviation from preliminary data, as before. We found *n* = 30 would result in 80% power to detect a 2.4-fold difference in the relative abundance of a transcript, while *n* = 60 would result in 80% power to detect 1.4-fold difference, assuming a balanced design.

## Trial Registration and Funding

The study is registered on clinicaltrials.gov with registration number NCT03246230.

The study is funded primarily by a grant received from the U.S. National Institute of Health; Grant number U19AI118608. These funders are not involved in study design, collection, management or analysis and interpretation of data, or publication of output. Additional funding is provided by the *Precision Vaccines Program*, supported in part by the Department of Pediatrics and Chief Scientific Office of Boston Children's Hospital.

The study will be conducted according to Declaration of Helsinki International Conference Harmonization Good Clinical Practice (ICH-GCP) and local ethical guidelines. Internal governance SOP's will be followed, and study-specific procedures developed by the study team.

## Ethical Considerations

Ethical approvals have been obtained for the core study in The Gambia from The Gambia Government/MRCG Joint Ethics Committee (Scientific Coordinating Committee number: 1513); for the validation study in PNG from the PNGIMR Institute Review Board (IRB number: 18.12) and the protocol has been approved by the PNG Medical Research Advisory Committee (IRB number 18.14). Ethical approval has also been obtained from the Boston Children's Hospital Institutional Review Board (IRB-P00024239).

## Participant Selection

### Study Sites

In the Gambia, 720 mother-infant pairs (60 per group) will be recruited through delivery rooms at one secondary level care institution; The Kanifing General Hospital and one primary health facility; The Banjullinding Health Center in The Gambia. In PNG, 80 mother-infant pairs will be recruited at Goroka Hospital, the only tertiary hospital in the Eastern Highland Province of PNG. Maternal infant pairs will be recruited within the first 24 h of the infants' life.

### Rationale for Selection of Participants

As infection is most prevalent in the newborn, the study will target this most vulnerable group to help improve our understanding of vaccine-induced cellular and molecular signatures associated with vaccine-induced correlate of protection i.e., anti-HBs Abs.

## Informed Consent

### Community Consent

Community consent will be sought prior to the commencement by providing information to individuals in the communities served by the participating health centers. Village heads and key opinion leaders in the communities will be engaged in large community meetings during which information regarding the study will be provided and opportunities given to ask questions, in line with previous practice at the MRCG and PNGIMR.

### Individual Consent

Women presenting for antenatal care in the 2nd or 3rd trimester of pregnancy will be approached at the antenatal care clinic and given information regarding the study using a detailed informed consent document. Individuals expressing interest in participating will then be provided with a copy of the informed consent document (Appendix) to take home to discuss with their partners or other significant decision maker in the family. Opportunities will be given to ask questions including the provision of contact details to allow potential participants to reach members of the study team after the clinic visit. Follow-up contact will then be made with each potential participant to verify spousal assent, as male members of the family are typically key decision makers in this setting. In addition, families will be asked to confirm if other members of the household need to assent including grandparents of the child in question.

When the mother of the potential participant presents in labor or shortly following delivery, the consent signature will be obtained, and eligibility assessed. Written consent will be obtained from biological mothers of participants in each mother-infant pair only. The consent process will continue throughout the study with confirmation of willingness to continue participation at each contact.

## Future Use of Stored Samples

Written informed consent will also be sought for the future use of left-over biological samples for studies which would require approval by the relevant ethics committees.

## Inclusion/Exclusion Criteria

### Maternal

Healthy women delivering at term will be recruited into the study. Inclusion and exclusion criteria are as detailed below.

#### Inclusion

Reported gestation of at least 37 weeks

Spontaneous vaginal delivery

Written informed consent.

#### Exclusion

Antibiotic use in the week prior to delivery

HIV or HepB positive—Rapid test done in labor or shortly after delivery

TB Diagnosis in mother or family member in the past 6 weeks

Severe intrapartum condition such as severe pre-eclampsia

Physicians assessment of high risk or previous contraindicative obstetric history such as multiple early neonatal deaths.

### Infant

Healthy term newborns will be recruited into the study. Details of inclusion and exclusion criteria are as follows:

#### Inclusion

Healthy, term infant as determined by medical history, physical examination, and judgment of the study physician.

Weight of 2.5 kg or greater at the time of enrolment.

#### Exclusion

Previous vaccination with any EPI vaccine

Major known congenital malformation

Apgar score <8 at 5th min.

Physician assessment of potential high-risk infant including significant risk factors for sepsis/morbidity (such as maternal symptoms suggestive of urinary tract infection in the peri-partal period), or macrosomia (birth weight above 4 kilograms).

## Randomization and Blinding

Following assessment of eligibility by the study physician, newborns will be randomly assigned to 1 of 12 groups in The Gambia (60 per group), and 1 of 4 groups in PNG (20 per group), using a computer-generated block randomization sequence. In The Gambia, the code will allow block randomization of 720 study participants into 12 groups, using random block sizes of 24 and 48. In PNG, block sizes of 8 will be used. Each study participant will be assigned sequential identification numbers (IDs) starting from 001 to 720 for The Gambia, and 1,001–1,080 in PNG. A check digit character will be augmented to each of these numbers to make up the participant IDs which will be generated using the Damm algorithm (https://en.wikipedia.org/wiki/Damm_algorithm; date accessed 08 November 2016). Here the check digit numbers 0–9 will be converted to letters A-H and J-K. The IDs for the Gambian cohort will be prefixed with the letter “G” which stands for Gambia. The randomization code will use the web application developed by the Statistics and Bioinformatics department at MRCG to generate the IDs including the check digit and prefix as detailed above (the app is available on https://stats.mrc.gm/chkdgt-id/). Randomization code for the PNG cohort will be generated at BCH using similar methods. Randomization groups will be printed on cards labeled with the participant identification number and allocated in the sequence in which the infants will be recruited. Cards will be sequentially allocated by clinical staff as participants are screened.

Due to the nature of randomized groups and difficulty of blinding to randomized vaccine groups, staff in direct contact with participants will be unblinded, while all laboratory staff at the sites will be blinded to participants assigned group throughout the study.

## Study Procedures

### Cohort Overview

The cohort overview is detailed in Table 1.

#### Screening

Following the informed consent signature and pre-test counseling, a detailed medical and pregnancy history will be obtained from each eligible woman. Women will be screened for Human Immunodeficiency Virus (HIV)-I and -II and HB-sAg using rapid diagnostic tests. Individuals positive for any of these antigens will be excluded from the study, offered post-test counseling and referred for care as appropriate. It will be essential to exclude HB-sAg positive mothers to ensure that infants born to these mothers receive HepB vaccine at birth to minimize transmission risk as recommended by the World Health Organization (WHO). The study team will ensure receipt of this vaccine within 24 h of life for all infants whose mothers tested positive for HepB, but these infants will remain excluded from further study procedures. Similarly, infants born to HIV-positive mothers will be excluded and referred for further management. Inclusion of HIV-exposed or positive infants could impact on the biological signatures. Each newborn infant will then undergo a full newborn exam to rule out obvious congenital anomalies and other signs of ill health.

#### Enrolment

Eligible mother-infant pairs will be randomized to one of 12 or 4 groups in The Gambia and PNG cohorts, respectively, and study procedures carried out as detailed below.

#### Follow-Up

The mother's participation will end following this initial visit while the infant will be followed up longitudinally over a 5-months period as dictated by the study design detailed in Table 1.

#### Termination

Participation in the study will be completed 1 month after the primary infant series vaccination, corresponding to 1-month post the 4th dose of HepB vaccine, or following the week one follow-up visit for infants in the delayed vaccine group (Groups 10 to 12) in The Gambia. Participation in PNG will be complete after the second visit for PNG; Table 1).

#### Early Termination

Infants will exit the study prior to the above if consent is withdrawn; the study team can no longer reach the participant's caregiver and/or caregiver does not present for a visit at the clinic (lost to follow-up); the participant moves out of the study area; or the infant dies.

#### Discontinuation Criteria

Study participation could also be discontinued for individual participants if in the judgement of the investigator participation poses an additional risk to the participant other than the already anticipated minimal risk. The study could also be discontinued following assessment by the Ethics Committee that the study posed significant unacceptable risk to the participants based on safety signals.

#### Strategies to Enhance Retention

To enhance participant retention and adherence to follow-up schedule, a field calendar will be set up which automatically generates the follow-up schedule in REDCap [a browser-based, metadata-driven electronic data capture (EDC) software and workflow methodology for designing clinical and translational research databases (https://www.project-redcap.org)], following enrolment. Each participant will be assigned to a field worker on the day of enrolment. This individual will keep a record of the follow-up schedule in their logbook. In addition, daily follow-up calendars will be printed from REDCap by field supervisors to ensure a double check for follow-ups. As an additional check, the clinician will maintain a field diary on which visits can checked off as they occur. The responsible field worker will call the participant to remind him/her of the scheduled visit at least 24 h prior to the visit (first week of life visit) or the weekend prior to the scheduled visit and again 24 h prior to the visit (subsequent visits). Participants will be picked up by a study vehicle for the first week of life visits to ease logistics during a time when mother and infant will be recovering from the birth process. Participants will be encouraged to share travel plans with the responsible field worker as soon as possible to allow for rescheduling of visits.

### Clinical/Laboratory Procedures

#### Blood Samples

The following samples will be collected as detailed in Table 1:

Two milliliters (ml) of blood will be collected at two time points within the first week of life, as described above. Immediately following blood draw into sodium heparinized tube (BD Vacutainer^R^, Cat. No. 368884), 200 μl of blood will be pipetted into a tube containing 552 μl/ ucl of Paxgene ^R^ fluid (BD Biosciences, Cat. No. 762165) for RNA stabilization in preparation for later sequencing. The remaining sample will be transported to the laboratory within 4 hours of collection along with RNA sample. Whole blood will be centrifuged to separate plasma. Plasma will be separated into 4 × 100 μl aliquots for onward shipment to analytical laboratories. Homogeneity of plasma aliquots will be ensured by first separating the total volume, mixing using micro pipetting, and subsequently separated into respective aliquots. Next, 900 μl of 1:1 whole blood and RPMI Medium 1640-GlutaMAX^TM^-I (Gibco^R^, Cat. No. 72400-047) will be aliquoted for immunophenotyping using the SMARTube system. Lastly, the remaining blood will be pelleted to obtain white blood cell. All fractionated samples will be stored at −70°C while awaiting and during shipment to endpoint laboratories.

Two additional samples of 3 ml each will be collected 30 days following enrolment and at Day 128 post enrolment which corresponds to 28 days post the 4th dose of HBV to measure anti-HBs Ag; a cell mediated immunity (CMI) assay will also be conducted using these samples. All samples will be collected by drip method into commercial tubes heparinized with sodium heparin (BD vacutainers).

Cord blood samples will be collected from a random subset of participants determined by logistics; where the mother has signed consent prior to delivery of the placenta and the infant is subsequently found eligible for enrolment. A total of 20 pristine cord blood samples will be targeted for collection at each site.

#### Rectal Swabs

Rectal swabs will be collected at the first study visit (within 24 h of life) if the child has passed meconium, and subsequently during the second visit (24 h, 72 h, or 7 days post first study visit) and at Day 30 post-enrolment to enable exploration of the infant microbiome. The swabs (FLOQSwabs (Copan, Cat. No. 608CS01R) will be collected using standard swabs and placed directly in 1 ml of guanidine thiocyanate transport media. They will be stored at −70°C before and during transport to analytical laboratories.

#### Vaccinations

Infants will be randomized into 1 of 4 groups (each split into 3 further subgroups to reflect day of second blood sample collection in The Gambia), to receive vaccinations as detailed in Table 1. Although not routinely recommended in PNG, the infants at this site will also receive bivalent oral polio vaccine within the first week of life as part of our efforts to harmonize schedules.

All subsequent childhood vaccines will be given in line with the World Health Organization (WHO) recommended EPI schedule, including 13-valent pneumococcal conjugate vaccine, rotavirus vaccine, Pentavalent vaccine containing diphtheria, whole cell pertussis, tetanus toxoid, HepB and *Haemophilus influenzae* type b antigens, oral polio vaccine and inactivated polio vaccine, as detailed in Table 1. Of note, the routine schedule in The Gambia involves vaccinations at 2, 3, and 4 months of age beyond the birth doses (Supplementary Table 1A), while that of PNG is 1, 2, and 3 months (Supplementary Table 1B). In an effort to harmonize between the two study sites, the WHO schedule of 6, 10, and 14 weeks will be utilized at both sites, and the rotavirus vaccine (Rotarix), not routinely given in PNG, will be administered. While there is heterogeneity between vaccines formulations routinely used in the two countries, vaccines from the same manufacturers will be utilized for both sites. The routine EPI schedules for both countries are detailed in Supplementary Tables 1A,B.

The specific vaccines which will be utilized for the study are detailed in Tables 2, 3.

**Table 2:** Vaccines to be studied in EPIC-002.

**Vaccine**	**Acronym**	**Source**	**Type**	**Route**	**Adjuvant**
Hepatitis B vaccine	HepB vaccine	SIIL	Sub-unit	IM	Alum
DwPT-HepB-Hib (HepB component)	Pentavalent vaccine	SIIL	Killed/toxoid	IM	Aluminum phosphate
Bacille Calmette–Guérin	BCG	SIIL	Live	ID	Self-adjuvanted live attenuated vaccine

*Ab, antibody; ID, intradermal; IM, intramuscular; EPI, WHO Expanded Programme on Immunization; DwPT-HepB-Hib, Diphtheria, whole cell pertussis, tetanus, Hepatitis B and Haemophilus influenza type b vaccine; SIIL, Serum institute of India Limited*.

**Table 3:** Other vaccines to be received during the EPIC-002 study.

**Vaccine**	**Acronym**	**Source**	**Type**	**Route**
Oral polio vaccine	OPV	SIIL	Killed	Oral
Inactivated polio vaccine	IPV	SIIL	Live	IM
13 -valent Pneumococcal Conjugate Vaccine	PCV13	Pfizer	Killed	IM
Rotavirus vaccine	Rotarix	GSK	Live	Oral

*ID, intradermal; IM, intramuscular; EPI, WHO Expanded Programme on Immunization; SIIL, Serum Institute of India Ltd.; GSK, GlaxoSmithKline; IPV, Inactivated polio vaccine; OPV, Oral polio vaccine; PCV13, 13-vallent pneumococcal conjugate vaccine*.

#### Laboratory Assays

The following assays are planned on samples collected (Supplementary Figure 1):

##### Transcriptomics

RNA Sequencing (RNASeq) at the University of British Columbia (UBC) Vancouver to assess the changes in gene expression in the blood samples collected within the first week of life.

##### Proteomics

Plasma samples will be subjected to nanospray liquid chromatography tandem mass spectrometry (LC-MS/MS) at Boston Children's Hospital (BCH) for protein identification and quantification by liquid chromatography mass spectrometry.

##### Flow cytometry

Flow cytometry will be run by UBC/TKI to analyze the changes in cell composition in the samples collected within the first week of life.

##### Basal plasma cytokines

Plasma samples will be analyzed utilizing multiplex cytokine assays to quantify cytokine and chemokine plasma concentrations across the first week of life using a custom multiplex kit (Milliplex MAP Kit: Human Cytokine/Chemokine Magnetic Bead Panel HCYTMAG60K-PX41) at BCH.

##### Cell mediated immunity (CMI)

Assays will be carried out at MRCG to assess the antigen-specific CD4 T-cell response following HepB and BCG vaccines for the Gambian cohort only.

*In vitro* modeling of vaccine responses using cord whole blood assays (WBAs; at MRCG and BCH) and microphysiologic tissue constructs (TCs) will be conducted at BCH.

##### Microbiome

Fecal (stool) microbiome composition and functional potential will be determined over the first month of life via 16S amplicon sequencing as well as metagenomics on selected samples to capture changes in association with vaccine responses.

##### Metabolomics

Plasma metabolomics will be analyzed using Metabolon's HD4 platform LC-MS/MS for identification of global plasma metabolites.

##### Epigenetics

To compare differential gene methylation patterns (epigenetics) following vaccination genome-wide changes in the epigenome will be assessed via the Infinitum Methylation EPIC bead array. The analytical laboratory for this assay is yet to be determined.

#### Sample Management

Sample management and tracking will use ItemTracker; a sample tracking and management system (http://www.itemtracker.com) to track samples from the point of collection to the analytic laboratories by scanning a sample-specific barcode which carries a unique sample identifier (visit ID).

## Safety Considerations and Oversight

### Risks and Benefits

We recently reported major cellular and molecular changes which occur over the first week of life demonstrating a dynamic baseline (17). For this reason, we will have a delayed vaccination group in order to distinguish basic immune ontogeny signatures from vaccine-induced changes.

To minimize risks, we will carefully screen mothers and infants to avoid any risk for the infants included in the delayed vaccination group by the following measures: (a) only newborns to HB-sAg negative mothers will be included; (b) any women with history of tuberculosis (TB) or TB contact will be excluded; (c) BCG vaccination is recommended for any time before 3 months of age and infants within the delayed vaccination group will be vaccinated within 10 days of birth. There is indeed evidence to suggest that delayed BCG vaccination may actually be beneficial (30). The continued recommendation for birth doses of the vaccine is to ensure coverage and avoid missed opportunities for vaccination during contact with a health facility. Polio vaccine is recommended at birth in The Gambia and will be delayed by a maximum of 10 days for all infants in our cohort. This infection has not been diagnosed in The Gambia for over 20 years. The risk posed from this slight delay is therefore considered minimal.

Overall, the Ethics Committees have determined that the study to pose minimal risk to participants, consisting of transient pain when drawing blood and possibly mild discomfort during the rectal swab. Rare complications could include bleeding from or inflammation/infection of puncture sites.

There are no direct benefits for the individual participants other than access to care at the research facility allowing for closer surveillance and higher likelihood of detecting illness early. Participants excluded due to maternal HepB positivity will have the added benefit of accessing the HepB vaccine within 24 h after birth from the study team. Despite being recommended by WHO, this practice is not yet uniformly carried out in many low-income countries including The Gambia and PNG, and largely depends on whether the child is born during working hours vs. the weekend and/or late night. In addition, in The Gambia vaccinations given after the tradition of infant naming at the age of 7 days of age is a common local practice also contributing to delayed vaccinations in the real-life setting.

All costs related to medical care of participants will be covered by the study for the duration of the study. Institutional insurance for participants exists for all research projects conducted at the MRCG Unit. Sponsors cover insurance for study participants at the site in PNG.

### Safety Oversight

Since the study infants are enrolled at an age at which they are particularly prone to illness, including infections, rigorous clinical oversight will be ensured. Oversight for participant safety will be provided by the lead study pediatrician with support from a local safety monitor—a pediatrician with experience caring for children at this age who could provide input as an independent party, in The Gambia, and a pediatrician affiliated with Goroka Hospital in PNG.

**Figure 2:**
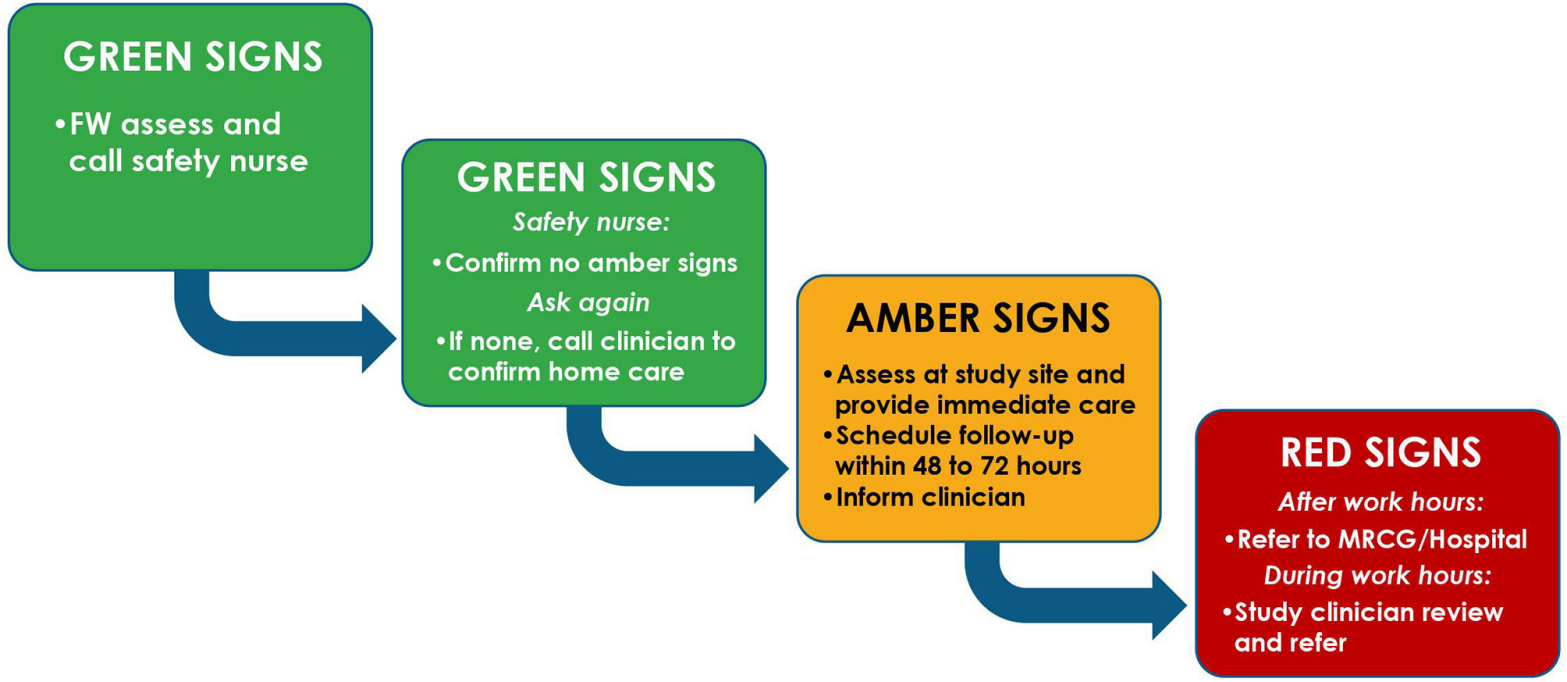
Field algorithm for management of intercurrent illness during the EPIC-002 study. Green, amber and red signs are as defined in Supplementary Table 2. FW, Field worker.

Field staff will conduct a home visit during the first week of life to evaluate for sick infants, such that all participants will have a total of 2 visits (home or clinic) prior to day 7 of life. Participants will also receive phone numbers at the point of enrolment to enable them reach study staff in the event of emergencies/concerns. Field staff trained to identify danger signs will utilize an escalation system where clinical signs are categorized into green (low risk), amber (intermediate risk), or red (high risk) as detailed in Supplementary Table 2 and Figure 2 to ensure prompt care for intercurrent illnesses.

## Data Handling, Records, and Analysis Platforms

In The Gambia, all data will be collected using electronic case report forms (CRFs) with built-in quality checks designed on REDCap except for consent forms and certified copies of medical records. Data capture using this platform could be done online or offline to allow entry in settings where internet access is suboptimal. In PNG, data will first be collected using paper CRFs before entry into an electronic REDCap database. Data will be prioritized into three Tiers: Tier-1 data considered essential to accomplish primary study objectives; Tier-2 data considered potentially useful for identifying confounders which might influence analysis of the primary study questions and to address secondary study questions; and Tier-3 data considered additional exploratory data. Data will be checked for congruency and completeness at the site and quality control (QC) checks will be completed by data assistants and a data manager at MRCG and PNGIMR. Queries will be generated and resolved by responsible staff, following which data forms will be locked by delegated personnel to prevent inadvertent changes. This will be followed by data extraction prioritized from Tier-1 to−3, and data will be sent securely to the centralized Data Management Core (DMC) at BCH. Further quality assurance (QA) processes will be performed by the DMC and queries raised to allow resolution of discrepancies/clarify unclear details prior to data sharing across the project. Following this process for all tiers of data, the database will be locked, and data downloads will be restricted to read-only access.

The DMC will use an innovative operational structure for data curation and integrative analysis. The digital backbone of the DMC will leverage an institutional partnership with Amazon Web Services (AWS) to provide secure dedicated cloud-based access and computational tools to research programs based at BCH. This will allow for controlled and timely updates of key infrastructure components, resources, and policies.

## Confidentiality

All electronic data capture will be performed using secure password-protected encrypted devices. Papers records including consent forms, and medical records will be stored in locked cabinets in secure locations with restricted access at the study sites.

## Quality Control and Quality Assurance

Written Standard Operating Procedures (SOPs) and Study Specific Procedures (SSPs) will be developed and implemented for each activity. Staff engaged in the activity will be trained to carry out the specific procedure in accordance with the written SOPs and SSPs. Copies of these procedures will then be provided to each staff member for reference and additional copies will be located at each study site for ease of reference.

A number of general QC and QA measures will be observed for the study including: (a) having study staff work in pairs and cross check one another's procedures and entries, including checking all procedures and entries before marking as complete; (b) having senior team members validate procedures and entries carried out by junior members and conduct spot checks for ongoing work of junior team members; (c) disallowing any blank fields on completed forms to ensure that where data is not available this is clearly documented and not just an omission in entry; and (d) requiring transmittal logs for materials/samples transported from one location to another to ensure that these can be tracked to their current location at all times.

### Laboratory Quality Control Measures

Controls and standards will be run for each assay. In addition, to minimize batch effects related to laboratory assays, additional Universal Reference Standard (URS) samples will be included with each batch of samples to allow comparison or data normalization to minimize potential run-to-run or plate-to-plate variation. As a further measure to reduce batch effects and mitigate potential confounding of these effects across platforms, sample batches for all “OMIC” platforms will be aligned with the study block randomization as follows:

- Each sample batch will include two study visits per study participant to minimize variation across time points;
- Sample batches will be generated and assigned using 48 or 96 samples (i.e., 24–48 participants over two visits) per block, with equal representation of each of the 12 vaccine sub-groups within each sample batch;
- Each assay team will predefine criteria for quality control of each experimental sample and assay to ensure data quality.

The overall approach will be to have each endpoint laboratory define its own quality control (QC) procedures with additional quality assurance (QA) performed by the centralized Data Management Core at BCH.

## Dissemination Policy

All data will be uploaded to public repositories such as NIAID ImmPort, https://immport.org and Gene Expression Omnibus (GEO) and will be publicly available following submission of study output for publication.

Study output will be published by the study team adhering to IJMCE authorship criteria in peer reviewed journals. Results will be disseminated at conferences and stakeholder meetings.

Community feedback will be undertaken to disseminate study results to participating communities.

## Author's Note

This protocol has been prepared in keeping with SPIRIT guidance.

## Author Contributions

OL, TK, and BK conceived the study. OL, TK, BK, RH, HS, RB, PR, ST, and AO developed study design. OI and AB developed protocol draft and contributed to study design. OI, OL, TK, BK, CS, KS, AB, and AO development of protocol content. OI and BK provide clinical oversight and developed site clinical protocols and procedures. All authors critical review of protocol content and review of final draft.

## Conflict of Interest

The authors declare that the research was conducted in the absence of any commercial or financial relationships that could be construed as a potential conflict of interest.

## Abbreviations

Ab: Antibody
BCG: Bacille Calmette-Guérin
CMI: Cell Mediated Immunity
EPI: Expanded Programme on Immunization
EPIC-HIPC: Expanded Programme on Immunization Consortium- Human Immune Project Consortium
HBV: Hepatitis B virus
HepB: Hepatitis B
HB-sAg: Hepatitis B Surface antigen
Anti-HBs: anti-Hepatitis B surface antigen
LSHTM: London School of Hygiene and Tropical Medicine
MRCG: Medical Research Council Unit The Gambia
PNG: Papua New Guinea
PNGIMR: Papua New Guinea Institute of Medical Research
WBA: Whole Blood Assay.

## Appendices

1. Informed consent document version 3.1 of 16 Feb 2018 (MRCG) and version 1.0 of 19 September 2018 (PNG)
2. Algorithm for identifying danger signs among study participants adapted from National Institute for Health and Care Excellence (NICE) guideline.
3. Routine EPI schedule for The Gambia and PNG
4. Sample processing flow chart

In addition to the named authors on the title page, The EPIC Consortium includes:

Alec Plotkin, Amrit Singh, Amy Lee, Arnaud Marchant, Asimenia Angelidou, Benoit Fatou, Bhav Dillon, Bianca Dy, Bing Cai, Daniel He, Danny Harbeson, David Ferrari, Diana Vo, Ebrima Trawally, Adam Jeng-Barry, Modou L. Fofana, AruKumba Baldeh, Joseph Kanu, Elena Morrochi, Fatoumatta Cole, Fatoumatta Ceesay, Frederic Clement, Geert Leroux-Roels, Geraldine Masiria, Gerard Saleu, Giulia Conti, Jensen Pak, John-Paul Matlam, Ken Kraft, Kim-Anh Le Cao, Maren Smith, Matthew Pettengill, Mehrnoush Malek, Mitch Cooney, Njilan Johnson, Rebecca Ford, Reza Falsafi, Sam Hinshaw, Sarah Javati, Sebastiano Montante, Shun Rao, Sofia Vignolo, Susan Farmer, Travis Blikie, Tue B. Bennike, Wendy Kirarock, Erin E. Gill, Shun Rao, Miriam Wathuo, Zaineb Wurie.
