# Corrigendum: Clinical Protocol for a Longitudinal Cohort Study Employing Systems Biology to Identify Markers of Vaccine Immunogenicity in Newborn Infants in The Gambia and Papua New Guinea

**DOI:** 10.3389/fped.2020.610461

**Published:** 2020-11-17

**Authors:** Olubukola T. Idoko, Kinga K. Smolen, Oghenebrume Wariri, Abdulazeez Imam, Casey P. Shannon, Tida Dibassey, Joann Diray-Arce, Alansana Darboe, Julia Strandmark, Rym Ben-Othman, Oludare A. Odumade, Kerry McEnaney, Nelly Amenyogbe, William S. Pomat, Simon van Haren, Guzmán Sanchez-Schmitz, Ryan R. Brinkman, Hanno Steen, Robert E. W. Hancock, Scott J. Tebbutt, Peter C. Richmond, Anita H. J. van den Biggelaar, Tobias R. Kollmann, Ofer Levy, Al Ozonoff, Beate Kampmann

**Affiliations:** ^1^Vaccines and Immunity Theme, Medical Research Council Unit the Gambia at London School of Hygiene and Tropical Medicine, Fajara, Gambia; ^2^Precision Vaccines Program, Division of Infectious Diseases, Boston Children's Hospital, Boston, MA, United States; ^3^CIH LMU Center for International Health, Medical Center of the University of Munich (LMU), Munich, Germany; ^4^The Vaccine Centre, London School of Hygiene and Tropical Medicine, London, United Kingdom; ^5^Harvard Medical School, Boston, MA, United States; ^6^PROOF Centre of Excellence, Vancouver, BC, Canada; ^7^Department of Pediatrics, BC Children's Hospital, University of British Columbia, Vancouver, BC, Canada; ^8^Division of Medicine Critical Care, Harvard Medical School, Boston Children's Hospital, Boston, MA, United States; ^9^Department of Cardiology, Boston Children's Hospital, Boston, MA, United States; ^10^Wesfarmers Centre of Vaccines and Infectious Diseases, Telethon Kids Institute, University of Western Australia, Nedlands, WA, Australia; ^11^Papua New Guinea Institute of Medical Research, Goroka, Papua New Guinea; ^12^BC Cancer Agency, Vancouver, BC, Canada; ^13^Department of Medical Genetics, University of British Columbia, Vancouver, BC, Canada; ^14^Department of Pathology, Boston Children's Hospital, Boston, MA, United States; ^15^Department of Microbiology & Immunology, University of British Columbia, Vancouver, BC, Canada; ^16^Centre for Heart Lung Innovation, University of British Columbia, Vancouver, BC, Canada; ^17^Division of Respiratory Medicine, Department of Medicine, UBC, Vancouver, BC, Canada; ^18^Division of Pediatrics, School of Medicine, Perth Children's Hospital, University of Western Australia, Nedlands, WA, Australia; ^19^Broad Institute of MIT & Harvard, Cambridge, MA, United States

**Keywords:** markers, newborn, vaccine, immunogenicity, systems biology, OMICS

In the original article, there was an omission in the legend for **Figure 2** as published. **“FW” in the figure stands for Field worker**. The correct legend appears below.

**Figure 2** **∣** Field algorithm for management of intercurrent illness during the EPIC-002 study. Green, amber and red signs are as defined in Supplementary Table 2. FW, Field worker.

In the original article, there was a mistake in Table 1 as published. Column headings below groups 4, 5, and 6 should read BCG and not HepB. The corrected Table 1 appears above.

**Table 1:** Clinical cohort table for infants recruited in The Gambia (discovery cohort) and Papua New Guinea (validation cohort).

** 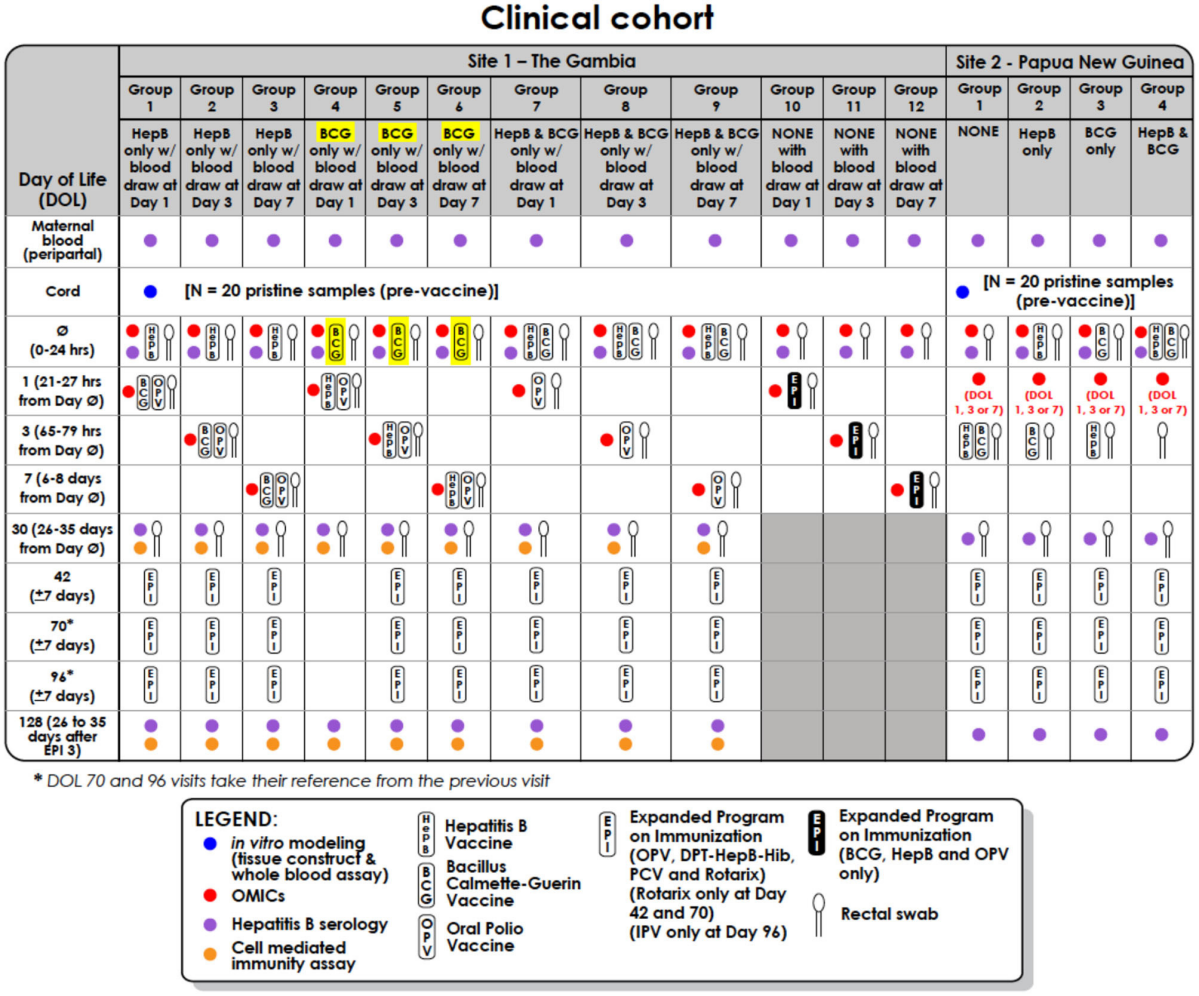 **

The authors apologize for this error and state that this does not change the scientific conclusions of the article in any way. The original article has been updated.

