# Supplementary material for: Clinical Protocol for a Longitudinal Cohort Study Employing Systems Biology to Identify Markers of Vaccine Immunogenicity in Newborn Infants in The Gambia and Papua New Guinea
Source: Front Pediatr. 2020 Apr 30;8:197. doi: 10.3389/fped.2020.00197 (PMC7205022; doi:10.3389/fped.2020.00197)
Supplement: Supplementary file 1 [file Data_Sheet_1.docx]

Supplementary Table 1a: Routine Expanded Program on Immunization schedule for The Gambia

| Age | Birth | 2 m | 3 m | 4 m | 6 m | 9 m | 12 m | 18m |
| --- | --- | --- | --- | --- | --- | --- | --- | --- |
| *Vaccine* |  |  |  |  |  |  |  |  |
| BCG | 🗸 |  |  |  |  |  |  |  |
| HepB vaccine | 🗸 |  |  |  |  |  |  |  |
| DTwP/HepB/Hib vaccine |  | 🗸 | 🗸 | 🗸 |  |  |  | 🗸 (DPT) |
| PCV13 |  | 🗸 | 🗸 | 🗸 |  |  |  |  |
| OPV |  | 🗸 | 🗸 | 🗸 |  |  |  |  |
| Rotavirus vaccine |  | 🗸 | 🗸 |  |  |  |  |  |
| IPV |  |  |  | 🗸 |  |  |  |  |
| Measles vaccine |  |  |  |  |  | 🗸 |  | 🗸 |
| Yellow fever vaccine |  |  |  |  |  | 🗸 |  |  |
| Men A vaccine |  |  |  |  |  | 🗸 |  |  |

Supplementary Table 1b: Routine Expanded Program on Immunization schedule for Papua New Guinea

| Age | Birth | 1 m | 2 m | 3 m | 6 m | 9 m |
| --- | --- | --- | --- | --- | --- | --- |
| *Vaccine* |  |  |  |  |  |  |
| BCG | 🗸 |  |  |  |  |  |
| HepB vaccine | 🗸 |  |  |  |  |  |
| DTwP/HepB/Hib vaccine |  | 🗸 | 🗸 | ✓ |  |  |
| PCV13 |  | 🗸 | 🗸 | 🗸 |  |  |
| OPV |  | 🗸 | 🗸 | 🗸 |  |  |
| IPV |  |  |  | 🗸 |  |  |
| Measles vaccine |  |  |  |  | 🗸 | 🗸 |

Key:

BCG – Bacille Calmette Guérin

OPV – Oral Polio Vaccine

PCV13 – 13-valent Pneumococcal Conjugate Vaccine

DTwP/HepB/Hib vaccine – Diphtheria, Tetanus, whole cell Pertussis, Hepatitis B and *Haemophilus influenzae* type b

HepB – Hepatitis B

IPV- Inactivated Polio vaccine

MenA – *Neisseria meningitidis* serogroup A (from March 2019 in The Gambia)

Supplementary Table 2: Algorithm for identifying danger signs among study participants.

|  | **GREEN- LOW RISK** | **AMBER – INTERMEDIATE RISK** | **RED- HIGH RISK** |
| --- | --- | --- | --- |
| **Colour of conjunctiva, lips, tongue, palms and soles of the feet** | Pink/ no change in infant’s colour | Pallor reported by care giver | Pale/Blue observed by study team |
|  |  | Yellowness of the eyes (check in natural light) | Yellowness of the eyes with any other red symptom |
| **Activity** | Awake and conscious | Decreased activity | Altered consciousness |
|  | Smiling and playing | Not smiling or playing | Appears acutely-ill to member of study team |
|  | Normal cry (not excessive or high-pitched) comforted by breastfeeding |  | Weak cry/ high-pitched cry/ continuous cry |
| **Respiratory** | Has only runny nose and cough. No other respiratory symptom or sign | Tachypnea  •RR> 60 (Neonate)  •RR> 50 (Infant) | Tachypnea RR> 70 (Neonate)  RR> 60 (Infant) |
|  |  |  | Chest in-drawing |
|  |  | SP0_2_ 90 to 95% | SP0_2_ < 90% |
| **Circulation and hydration** | Occasional vomiting 1 to 2 episodes per day | Vomiting 3 to 4 times in a day | Vomiting ≥ 5 times in a day |
|  |  |  | Greenish vomiting in a neonate |
|  | Few episodes of watery stool | Episode of watery stool 3 times frequency of normal bowel habit | Episode of watery stool ≥ 4 times frequency of normal bowel habit |
|  | Breastfeeding well |  | Poor feeding |
| **Other** | No fever | Low grade fever (37.5 to 37.9^o^C) in the absence of any red symptoms or signs | Low grade fever (37.5 to 37.9^o^C) with any red symptoms or signs |
|  |  | Moderate fever (38^o^C to 38.5 ^o^C) in the absence of any red symptoms or signs | Moderate fever (38 ^o^C to 38.5 ^o^C) in a neonate or with any red symptoms or signs |
|  |  | Fever following an immunisation visit | High temperature >38.5 ^o^C |
|  |  |  | Low temperature ≤ 35 ^o^C |
|  |  |  | History of convulsion or active convulsions |
|  |  | Abnormal body swellings |  |

*Adapted from National Institute for Health and Care Excellence (NICE) traffic light system for identifying serious illness in children; Management by remote assessment*

***Notes for field staff****:*

1. *Newborns may pass stool following each breast feeding (up to 8 bowel motions in 24 hours) and this is normal. To assess for diarrhoea in a newborn please confirm details with a clinician.*
2. *Where there is a report of diarrhoea always ask for presence of blood in stool.*
3. *Convulsions in a newborn may be subtle and include things like lip smacking, eye blinking and twitching.*

Supplementary Figure 1: Sample Processing flow chart

CMI, cell mediated immunity assay; PBMC, peripheral blood mononuclear cell; PPP, platelet-poor plasma; WB, whole blood; WBC, white blood cell.
